# What has been the contribution of the first Global Fund grant (2003-2006) to malaria control and health system strengthening in Timor-Leste?

**DOI:** 10.1186/1475-2875-9-S2-O23

**Published:** 2010-10-20

**Authors:** Joao Martins, Anthony Zwi, Paul Kelly

**Affiliations:** 1 School of Public Health and Community Medicine, University of New South Wales, Sydney, NSW, Australia, 2052; 2 Universidade da Paz, Dili, Timor-Leste; 3 Australian National University, ACT, Canberra, Australia, 0200

## Background

The Global Fund to fight against AIDS, Tuberculosis and Malaria was established in Geneva in January 2002 with the main objective of mobilizing resources from governments, donors, private sector and individuals to tackle HIV/AIDS, tuberculosis and malaria worldwide. Timor-Leste successfully obtained the first Global Fund grant with a value of nearly US $3 million in 2003 for a three-year program for malaria control. The grant was aimed at reducing malaria-related morbidity and mortality by 30% by the end of the implementation. Necessary structures required by the Global Fund to allow grant implementation were established.

## Method

A mixed methods approach was used to assess the impact of the grant implementation. The qualitative method employed in-depth interviews, group interviews, focus group discussions and observations. The quantitative method used the routinely collected morbidity data reported to the Ministry of Health.

## Results

**Figure 1:**
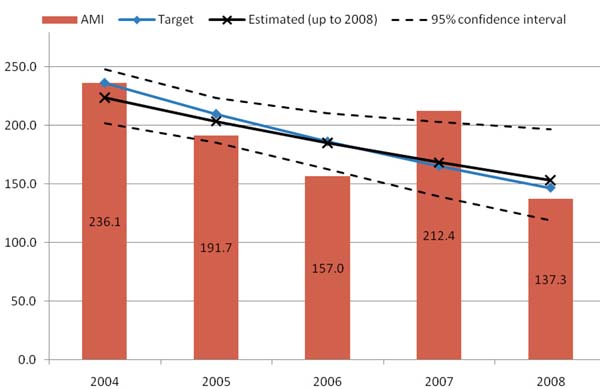
The AMI per 1000 in Timor-Leste 2004-2008.

Timor-Leste with the Global Fund grant managed to reduce malaria morbidity by around 10% but did not achieve the stated objective (Figure 1). However, the Global Fund contributed considerably to malaria control program establishment, strengthening control interventions and the health system in general. It also brought direct benefits to pregnant women and the community at large. The implementation was hampered by inadequate human resources, the rigidity of Global Fund rules, weak project management and coordination, and lack of appropriate support.

## Conclusion

Despite limitations, the grant was maintained until the agreed closing date. Considerable contributions to malaria control, health system and community have been made.
